# Examining Nutritional Vulnerability in an Under-Resourced Community in Northeastern Connecticut

**DOI:** 10.3390/nu18091353

**Published:** 2026-04-24

**Authors:** Xiran Chen, Daniela C. Avelino, Sydney K. Clements, Manije Darooghegi Mofrad, Xiang Chen, Michael J. Puglisi, Valerie B. Duffy, Ock K. Chun

**Affiliations:** 1 Department of Nutritional Sciences, University of Connecticut, Storrs, CT 06269, USA; xiran.chen@uconn.edu (X.C.); manije.darooghegi_mofrad@uconn.edu (M.D.M.); michael.puglisi@uconn.edu (M.J.P.); 2 Department of Allied Health Sciences, University of Connecticut, Storrs, CT 06269, USA; daniela_carolina.avelino@uconn.edu (D.C.A.); valerie.duffy@uconn.edu (V.B.D.); 3 Department of Philosophy, Political Science, and Geography, Eastern Connecticut State University, Willimantic, CT 06226, USA; clementss@easternct.edu; 4 Department of Geography, Sustainability, Community and Urban Studies, University of Connecticut, Storrs, CT 06269, USA; peter.chen@uconn.edu

**Keywords:** nutritional vulnerability, Web GIS mapping, liking-based Diet Quality Index, Short Healthy Eating Index, food security, SNAP participation

## Abstract

**Background/Objectives**: Nutritional vulnerability (NV) describes the interaction of diet quality, access to food, health status and socioeconomic factors and may differ between neighborhoods. Nevertheless, there is still a limited amount of evidence regarding local NV variations in contrasting resource landscapes. The purpose of this study was to operationalize NV in Windham, Connecticut and conduct an analysis of its spatial distribution and the differences between neighborhoods for NV and specifically diet quality. **Methods**: NV was measured with four indicators, including two diet quality measures (liking-based DQI and short food frequency-based sHEI), food security, obesity, and SNAP participation. Areas of vulnerable concentration were determined through spatial mapping. Indicators related to each other were measured by Spearman correlation. To compare the contrasting neighborhoods (resource-dense vs. resource-limited), contextual differences were studied and differences in NV indicators, sociodemographic and movement factors were compared with the help of chi-square tests. Diet quality measures were jointly examined for concordance (both measures low or high) and discordance. **Results**: Area-level comparisons showed significant differences in mobility-related and sociodemographic characteristics, including vehicle access and education level (*p* < 0.05). High diet quality (measure concordance) was reported by individuals living in high-resourced regions; low diet quality (measure concordance) by individuals in low-resourced regions. **Conclusions**: The NV Map illustrated focal patterns of vulnerability determined by the interplay of sociodemographic disadvantage and mobility-related limitations and not by distance to food resources. These results give practical spatial data to promote specific nutrition and resource intervention.

## 1. Introduction

The problem of nutritional disparities in the United States is being gradually recognized as a complex of social, economic and environmental aspects affecting dietary practices and health. From this perspective, the concept of nutritional vulnerability (NV) has been developed to reflect how the intersecting disadvantages restrict access and accessibility to healthy diets among people [1]. NV in this study is described as a combination of dietary inadequacy, food insecurity, risk of obesity, and reliance on nutrition assistance demonstrating that structural and socioeconomic obstacles raise nutrition-associated health hazards [2]. Families with long-term socioeconomic disadvantage, transportation constraints, and increased food costs prefer high-calorie, low-cost food items. These foods reduce the quality of diets and predispose to obesity, diabetes, and cardiovascular disease. These health outcomes are not merely the result of individual choices, but they occur due to broader structural limitations that overload resource-poor communities, which increases NV [1].

The existence of geographic disparities in the local food environment has been traditionally used to demonstrate how structural circumstances affect dietary habits and health hazards associated with nutrition. In NV, these spatial disparities offer one means through which socioeconomic and mobility restrictions restrain residents, thus their access to and consumption of nutritious foods. Areas that lack a full-range grocery store and have a large number of convenience stores and fast-food establishments tend to have a narrow assortment of low-energy, nutrient-poor foods [3]. Beyond low-access classifications of food deserts framing, studies on food swamps, or places with a high density of unhealthy food establishments compared to healthier food ones, indicate that obesity might be better predicted by excessive exposure to energy-dense food retail environments rather than by the absence of grocery stores [4]. The combination of economic strains, lack of transport services, and shortage of culturally suitable food establishments in these regions makes it difficult to keep a healthy diet amongst residents of these regions, aggravating the condition in NV [5]. Specifically, the availability of culturally specific foods—including traditional staple products and ingredients that are integral to the cultural foodways of ethnic minority and immigrant communities—can be limited. These foods are often available only in small grocery stores (e.g., bodegas), where prices may be higher [6], or they require long-distance travel to obtain [7], as the research team has observed in Northeast Connecticut. This may lead to a displacement of the healthier culture-based eating behavior with the preparation and eating of home meals in a traditional style [8]. These types of food-landscapes are not confined environmental peculiarities, but also they are indicative of the larger socioeconomic deprivation and transportation constraints that interrelate to influence how residents utilize and allocate food resources [3].

In addition to differences in the physical food environment, NV also relies on the uniformity of the food that households have access to and eat. Although food retail outlets are available in a community, limited money, time, and transportation may make individuals unable to access nutrient-rich foods, leading to fiber, vitamin, and mineral-deficient diets. These issues may be accompanied by food insecurity that makes NV higher in low-income households, together with these concerns [9]. Studies indicate that this is particularly the case in neighborhoods where grocery stores are distant, transportation systems are ineffective and most buyers use low-cost retailers [10].

Digital tools have the potential to help individuals navigate resources in a community and organizations to reach these individuals. Digital tools include online grocery order systems, online coupons and sales fliers, food delivery services, and SNAP-enabled online purchasing. Digital literacy is necessary to search, identify, interpret, and use the resources. Food and nutrition literacy supports identifying healthy food that meets health needs, especially on a limited food budget, and avoiding heavily marketed processed foods. Digital tools also are progressively more important to service providers and policymakers to enhance the organization and delivery of food-related resources [11]. Unfortunately, digital channels also can market processed or ultra-processed foods, such as on social media, which promotes their purchase and consumption [12]. For reaching vulnerable groups, most digital platforms are set up to provide the services and localize the resources without assessing the outcomes related to nutrition. Consequently, they do not combine people-focused measures such as quality of diet, food security and obesity.

With the development of food-access studies, researchers are starting to note the limitations of using geographic proximity to food retail outlets as the sole mechanism of understanding NV [13]. The geographic information systems (GISs), which are traditional, are usually utilized to map the places where food retailers are located and categorize the community food environments [14]. Such spatial tools have the potential to reflect trends in food, but they fail to capture the human behavioral dimension of NV, including eating patterns, mobility constraints and resource utilization. In several communities, opinions on safety, transportation availability, community attachment, and cultural inclinations by the people also influence food-access choices and not only distance views [15]. These revelations highlight the importance of going beyond the geography-based assessments to using NV research methods that are based on lived experiences of people.

NV is a multidimensional phenomenon by its nature and captures accumulated impacts of dietary, socioeconomic and mobility-related disadvantages, which often have geographical concentrations. Poverty and lack of walkability, accessibility to public transportation, and awareness of food resources frequently place communities in chronic poverty in the intersecting restrictions that reduce their food options [16]. This clustering is the reason why some communities have always demonstrated poorer dieting and greater prevalence of nutrition-related health issues. Previous studies have emphasized specific parts of these disadvantages, but there is a lack of literature that links several NV dimensions into a single spatial model to identify high-vulnerability neighborhoods and analyze those contextual factors that influence such trends. The gap requires analytical approaches that can show the intersection of dietary, socioeconomic, and mobility constraints within the specific neighborhood settings.

To address these gaps, this study employs a framework of community-level mapping of nutritional vulnerability (NV) in an underserved area of northeastern Connecticut. It is a combination of personal statistics on the quality of the diet, through the liking-based diet quality index (DQI) and the short Healthy Eating Index (sHEI) and the data on food security, obesity, and the utilization of food assistance. This aids in the identification of neighborhoods having high NV. The influence of sociodemographic and mobility factors on vulnerability is also considered by this study through the comparison of the neighborhood contexts. The NV Map is more than a simple creation of a tool; it is a spatial reference that connects individual measures with the resources of neighborhoods to present evidence to target resource allocation and nutrition interventions needed to address the individual needs and structural barriers [17,18]. Thus, this research study intends to characterize nutritional vulnerability (NV) and study its spatial distribution in the community.

## 2. Materials and Methods

### 2.1. Research Area

This study was carried out in Windham, Connecticut, the under-resourced community in northeastern Connecticut where socioeconomic disadvantages, transportation accessibility, and food retail opportunities are extremely different among neighborhoods. Such neighborhood-level disparities provide inequitable opportunities among residents to access and utilize food resources, which makes Windham an ideal location to study how NV is spatially distributed. The accessibility of full-service grocery stores is low in some parts of Windham, the coverage of public transportation is uneven, and the amount of mobility resources varies, which can influence the way residents obtain food and what they consume. The recent price rise and modifications of food assistance programs have burdened household budgets, which can result in a rise of low-cost and low-nutrition foods and a growing gap in NV disparities across the neighborhoods [19]. Stakeholder feedback has highlighted several community-related challenges, including limited transportation, insufficient availability of culturally suitable food options, and low awareness of existing local food resources; these findings further support the significance of geographically aggregated nutritional disadvantages [7]. These structural barriers are relevant to food preferences and availability of fresh and culturally favorable foods. Communities that are under-resourced with low access to healthy food are at risk of poor diet quality and greater health risks [20].

### 2.2. Data Sources and Aggregation

It recruited a convenience sample of Windham residents by means of in-person recruitment of local food pantries, libraries, community centers, and neighborhood events. The recruitment emphasized the residents of the lower-resource communities to enhance the sample that represents the populations with transportation and food access barriers. (BRANY IRB approval #24-230-910). A total of 285 respondents filled out surveys, of which 274 gave verifiable residential addresses to be incorporated into geographic aggregation. Diet-related indicators (DQI and sHEI) could be used with 196 of the participants and food security, obesity status (figure rating proxy), and SNAP participation with the entire sample. Individual vulnerability to neighborhoods was evaluated using these variables. TIGER/Line shapefiles (U.S. Census Bureau, 2024) were used to geocode and match the residential addresses with the census blocks in the town of Windham, CT. Data aggregation to the census-block level to improve confidentiality and analytic stability was then done following usual customary practices in spatial health research [21]. Continuous variables (DQI, sHEI) were represented using block-level means, whereas the categorical variables (food security, obesity classification, SNAP participation) were represented as proportions.

#### Variables and Indicator Framework

The Nutritional Vulnerability Map (NV Map) has four main indicators that highlight dietary, health, and socioeconomic dimensions of nutritional vulnerability.

Diet Quality—Two measures were used for improved assessment of the quality of the diet [22]. One is a liking-based Dietary Quality Index (DQI), a cognitively simple and validated index from reported liking of individual foods/beverages, averaged into food groups, weighted as healthy and less healthy groups, and finally averaged into an index [23,24,25]. The second is a short Healthy Eating Index (sHEI), which is computed on a short food-frequency survey [26]. Although the liking- and frequency-based diet quality indexes correlate [23,24], the discrepancy can denote structural, economic, and attitudinal constraints on individuals.

Food Security (FS)—To identify the stability of food access, the USDA six-item short form [27] was used to measure food security and aggregate it on the block level. The scores were classified as low, medium and high food insecurity, with the higher scores showing the highest food insecurity.

Obesity Rate (OB)—The Stunkard Figure Rating Scale (FRS) was used as a validated visual measure of obesity status (a proxy of BMI) in community and resource-constrained environments where direct anthropometric data measurement is not feasible [28]. The FRS has practical and psychological benefits in community-based research, especially among low-income groups, in that it reduces respondent burden, limits the discomfort related to reporting of weight-related scales, and enhances participation in those areas where clinical measurement is challenging to gain [29,30]. The participants were asked to choose the figure that they considered to be similar in size to their body, and the concept of obesity was determined by using established cutoffs. The rate of obesity was then determined as the ratio of the participants who were obese in each census block.

SNAP Participation Rate (SNAP)—The eligibility of SNAP participation was determined as a percentage of those in each block who self-reported to participate in the Supplemental Nutrition Assistance Program [3,31].

A combination of these four indicators reflects the multidimensionality of nutritional vulnerability, as it combines the quality of the diet, access to food and health outcomes, and socioeconomic support [32,33]. The combination of these variables into a single spatial framework makes it possible to identify regions where the nutritional and socioeconomic disorganization cross [13,34].

### 2.3. Data Processing, Mapping and Visualization

The GIS data of all spatial data were manipulated in ArcGIS 3.3 (Esri, 2024) with the coordinate system of NAD 1983 StatePlane Connecticut (Feet) [35]. The base geographic boundaries were provided by TIGER/Line shapefiles (U.S. Census Bureau, 2024). To assist spatial interpretation of NV, geocoded survey data were combined to census-block polygons using spatial attributes. The tabular data were then compared with the aggregated values to make sure that the data are consistent [36].

Aggregated indicators were combined into the NV Map as a part of the Smart Nutrition Program. It was not an independent product but a frame of analysis that used a series of nutrition-related indicators at the census-block level. It assisted in determining the neighborhoods with multiple dimensions of NV concurrency and offered a spatial background to subsequent statistical measurement and comparison of neighborhoods [13,34].

Symbology, layout of legends and the scale of labels were also standardized among all figures [37]. Map used a six-class Natural Breaks (Jenks) scheme in order to enhance the interpretability of census blocks that included unequal data [38]. ArcGIS Pro (version 3.3, Esri, Redlands, CA, USA) was used to create the statistical maps, whereas ArcGIS Experience Builder was used to create interactive web visuals to allow the use of non-technical people [39]. This method enabled the community partners to read and understand patterns in space without the knowledge of GIS.

This study’s emphasis was on making NV spatial patterns easy to interpret instead of being more interactively technical and visually complex. The approach simplified the process of converting the aggregated survey data into a spatial representation that could be used in community planning and policy debates [37,40,41]. The processing processes were completely documented to make the workflow reproducible and transparent [42].

### 2.4. Data Analysis

#### 2.4.1. Spearman Correlation Analysis

As the dataset contained both continuous and categorical variables, and the continuous variables were not normally distributed, Spearman rank-order correlations were employed to analyze the relationships among the four primary indicators, including two measures of diet quality (DQI and sHEI) [43]. All the calculations were done on an individual level and with IBM SPSS Statistics (version 29, IBM Corp., Armonk, NY, USA); the significance was taken at α = 0.05 [33]. Among the study sample, 196 participants reported diet-quality data (DQI and sHEI); all participants reported the data on food security, obesity status, and SNAP participation.

The monotonic diet quality, food security, and obesity classification and SNAP participation associations at the individual level were described by the Spearman analysis. The associated correlation table provides a descriptive background that explains the interaction of these indicators.

#### 2.4.2. Neighborhood-Level Comparison

As a supplement to block-level visualization and correlation analysis, researchers used a comparison at the neighborhood level to identify how demographic factors, resources of mobility, and food environments correspond to nutrition-related outcomes. A map-guided selection of two neighborhood areas was used: researchers analyzed separate census blocks using the NV Map and combined these blocks into two places that would show some clear differences in the food-access situations and NV trends. There were two selection criteria used:

1. Contrast in food-access situations, which refers to variations in the density and spatial structure of food-related commercial services.
2. Obvious outliers in NV patterns, signified by contrary or conflicting clusters on the NV Map (e.g., high SNAP participation in low diet quality and low SNAP participation in medium diet quality).

Based on these criteria, one resource-dense commercial corridor and one resource-limited residential area were identified for comparison. The participants were categorized in each area based on their verified residential address. Chi-square tests were used to analyze differences in groups in terms of diet-quality categories, food security level, obesity status, SNAP participation, and vehicle access (included as a contextual mobility factor as opposed to a core indicator). Continuous dietary indicators (DQI and sHEI) were scored with the median of the samples to compare the results. When the cell counts were lower than five as expected, Fisher’s exact test was applied, and the statistical significance was established at α = 0.05.

In the description of neighborhoods, researchers made a distinction between retail food outlets and community food resources. According to the USDA ERS Food Access Research Atlas, GIS-based measures of food access with a focus placed on distance to the types of stores, supermarkets, supercenters, or large grocery stores were considered. Retail outlets that failed to conform to this definition like convenience stores and small groceries, were not included as grocery stores but were classified as a part of a larger retail food environment. Food resources such as food pantries, soup kitchens, libraries, and community centers, where food programs were offered, were considered non-retail access channels and addressed as possible recruiting locations and service infrastructure instead of being regarded as being on the same level as full-service grocery retail [44]. Neighborhood contexts were further analyzed in terms of significant disparities in demographic composition, e.g., age, race and education level, that were used to provide further comparisons of diet quality on the neighborhood level.

#### 2.4.3. Neighborhood-Level Diet Quality and Demographic Analysis

Information from both diet quality indexes was used jointly to compare diet quality between the neighborhoods. Accordingly, each index (DQI and sHEI) was split at their respective median values to form concordant and discordant groups [23] as follows:

Concordant groups:

1. Low sHEI/Low DQI (consistent low dietary quality).
2. High sHEI/High DQI (consistent high dietary quality).

Discordant groups:

3. Low sHEI/High DQI (preference without behavioral translation).
4. High sHEI/Low DQI (potential misreporting of consumption, trying to improve dietary consumption).

Based on survey-reported address, participants were grouped into two neighborhood contexts: a resource-dense commercial area and a resource-limited residential area. The research team assessed for frequency differences in the four dietary groups for each neighborhood with the chi-square statistic.

Neighborhood-level differences were examined for demographic characteristics (age, race, and education) and illustrated by grouped bar charts. Proportions were calculated as a distribution of the total sample in each demographic–neighborhood grouping and reported as descriptive measures.

Binary logistic regression models were used to examine the association between dietary concordance patterns and neighborhood context, adjusted for sociodemographic variables. Neighborhood context (resource limited = 0; resource rich = 1) was the dependent variable, and Low sHEI/Low DQI was the reference group. Models were first run unadjusted to obtain crude estimates and then adjusted for age group, race, and education. Race was defined as White or non-White due to sample size.

In concordance-based regression analyses, only those who had complete sHEI and DQI data were included in the analysis. The results were then reported as odds ratios (ORs) with 95% confidence intervals (CIs).

## 3. Results

### 3.1. Overview of NV Map Development

The NV Map represents a community-level disparity of diet quality, food security, obesity, and food assistance program usage in Windham, CT. The map is a compilation of four indicators at the census-block level, including diet quality (DQI and sHEI), food security, obesity, and SNAP participation, which provides a summary of the localization of nutritional and socioeconomic disadvantages.

**Figure 1:**
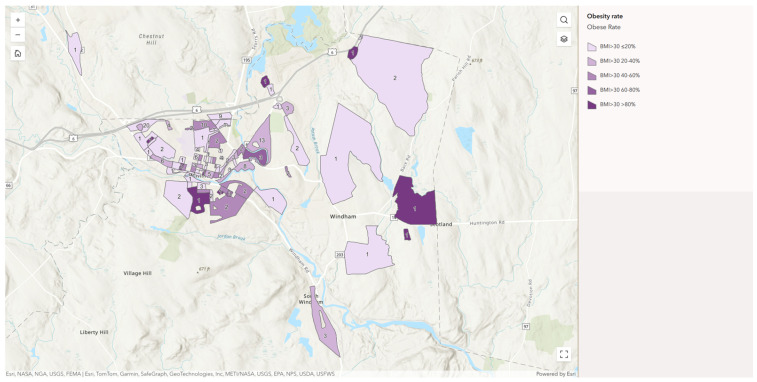
NV map displaying spatial patterns of obesity rates, food access, and health indicators in Windham, Connecticut. To enhance map readability and avoid visual clutter, this section only utilizes the obesity rate map layer for demonstration purposes, where different shades of purple represent the obesity levels within each neighborhood. Food security level was determined according to the USDA six-item short-form food security module, scores range from 0 to 6, with 0–1 being food secure, 2–4 being low food security, and 5–6 being very low food security; SNAP participation rate represents the proportion of households enrolled in the Supplemental Nutrition Assistance Program (SNAP) and was divided into four quantile-based categories (<20%, 20–40%, 40–60%, >60%) to reflect local variation; obesity rate (BMI ≥ 30) was calculated from community health survey data using CDC definitions and categorized by Natural Breaks (Jenks) to capture local disparities (20–40%, 40–60%, 60–80%, >80%); short Healthy Eating Index (sHEI) was computed based on the U.S. Department of Agriculture’s Healthy Eating Index framework, adapted to a shortened version validated in population-based dietary research (scores 0–100) and grouped into quartiles (<25, 25–50, 50–75, >75); and diet quality index (DQI) was calculated following the U.S. Department of Health and Human Services (HHS) and U.S. Department of Agriculture (USDA) dietary guideline scoring approach, with classification intervals (39.14–53.21, 53.21–63.38, 63.38–70.53, 70.53–80.00) derived using the Natural Breaks (Jenks) method in ArcGIS.

As shown in Figure 1, the NV Map provides a census-block-level view of the variation in diet quality, food security, obesity, and food-assistance program participation in Windham [45]. Among dietary quality indicators, the sHEI uses a 0 to 100-point scale for scoring, while the DQI employs a ±250-point scale. Higher scores indicate superior dietary quality [26,46]. The USDA six-item short-form module is used to measure food security based on the degree of food security of households. The Figure Rating Scale is used to estimate the status of obesity as opposed to direct anthropometric evaluation. Participants were asked to select the image that best represented their present body size, and the determined choice was further categorized into underweight, normal weight, overweight or obese based on previously established BMI-validation guidelines [47]. This has been found to be an effective proxy measure of weight conditions in community-based research where the direct BMI measurement is not achievable. The SNAP participation is reported as the percentage of households who self-reported that they are currently enrolled in the Supplemental Nutrition Assistance Program.

The NV Map in this research is descriptively employed to aid the subsequent statistical and neighborhood-level analyses and not as a result of analysis. The distributions of the various dimensions of NV as depicted in the map depict the distributions of the dimensions of the neighborhoods.

### 3.2. Spearman Correlation Results

Spearman rank-order correlations were initially analyzed between the four key indicators to support the construction of the Nutritional Vulnerability (NV) Map (Table 1). The SNAP participation had a moderate positive correlation with the rate of obesity (ρ = 0.27, *p* < 0.01), indicating that participants receiving SNAP benefits were more likely to have higher obesity rates. The sHEI score had a positive correlation with the food security score (ρ = 0.22, *p* < 0.01), indicating that higher food insecurity (i.e., higher food security scores representing low or very low food security) was associated with higher sHEI scores but not with DQI.

Even though most relationships between indicators were not statistically significant, the directional trends indicated that lower diet quality was linked with increased SNAP enrollment and obesity. The patterns demonstrate the multidimensionality of NV and give empirical evidence to the need to incorporate several indicators into the mapping framework.

### 3.3. Neighborhood-Level Comparison: Resource-Dense Area vs. Resource-Limited Area

**Figure 2:**
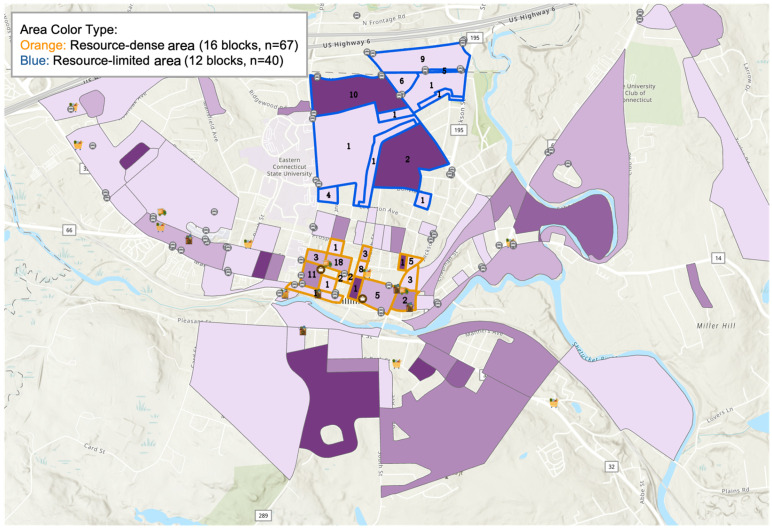
Boundary map of the two selected neighborhood areas (resource-dense area and resource-limited area). The resource-dense area represents the core commercial center of Willimantic and includes the highest concentration of services, transit stops, and grocery options. The area forms a quadrilateral defined by four major streets, encompassing the town’s walkable retail and service corridor. The resource-limited area is positioned near the university corridor north of downtown Willimantic. This zone consists largely of residential areas serving university populations and is framed by four major streets (U.S. Highway 6, Jackson St, High St, Lewiston Ave). The two neighborhood areas are geographically proximate (approximately 0.5 miles (~780 m) between representative downtown and university-corridor reference points), but the resource-limited area includes blocks that extend beyond the 0.5-mile urban food-access distance threshold to the nearest full-service grocery retail. This section utilizes an obesity rate map layer for demonstration purposes, where different shades of purple represent the obesity levels within each neighborhood. The numbers displayed within the selected areas indicate the aggregated count of participants at the neighborhood level.

As shown in Figure 2, a comparison of neighborhood characteristics of the resource-dense area (marked with an orange line) and the resource-limited area (marked with a dark blue line). The present comparison explores the relationship between demographic factors, mobility resources, and food environments and the nutritional outcomes. Each of the outlines contains all the census blocks that are used in the comparison.

- Resource-Dense Area: The main business district of Willimantic, which has the greatest percentage of grocery stores, transportation and mixed-retail locations. It creates a pedestrian business street enclosed by four large streets.
- Resource-Limited Area: This is an area located along the university corridor, and it is north of downtown Willimantic. It is surrounded by U.S. Highway 6, Jackson Street, High Street, and Lewiston Avenue, and it has no grocery stores.

#### Demographic Characteristics

To further examine the association between neighborhood context and NV, a descriptive comparison was made between two similar but structurally different regions within the study area: a resource-dense area and a resource-limited area. Table 2 presents the primary characteristics of participants in each area. Together, these variables give an idea of the differences between neighborhoods in terms of their population structure, mobility resources, and nutrition-related conditions and present significant context to more specific comparisons that will be carried out.

According to Table 2, the age difference between resource-dense and resource-limited areas was sharp (*p* < 0.001). A total of 72.5% of residents in the resource-limited area are young adults in the age range of 19–34, which corresponds to the student-oriented neighborhood around the university. Comparatively, the resource-dense area has a more diverse age structure. There is also the difference in the racial composition (*p* < 0.05). The resource-dense area has the majority of the White residents (53.7%), whereas the resource-limited area has a higher percentage of residents that are of other races (42.5%; other races include American Indian, Asian, Native Hawaiian or Other Pacific Islander, and Puerto Rican) and a slightly higher percentage of Latino/Hispanic residents.

The most distinguished difference between the two areas is the vehicle access (*p* < 0.001). A total of 61.2% of households in the resource-dense area do not own a driveable vehicle; this may be because the area is walkable with a downtown and has a powerful transit system. There is 80% vehicle ownership in the resource-limited area, which implies that a lot of the population, probably students or young tenants, is relying on their own vehicles since internal bus stops do not come to this area.

Diet quality scores (DQI and sHEI) did not significantly differ in both regions, but their trends were different. There was also a difference in the patterns of BMI, with resource-limited areas having 10% higher rates of underweight respondents and obesity prevalence being reached only by 15%. There were no differences in food security, though the food-secure population was somewhat better in the resource-limited area (45.0 vs. 32.8%).

The difference in participation in food assistance was quite big in the two areas (*p* < 0.001). A total of 61.2% of households living in the resource-dense area were enrolled in at least one food-assistance program. Conversely, 77.5% had none of the programs in the resource-limited area.

### 3.4. Variation in Diet Quality and Demographic Characteristics Across Neighborhoods

**Figure 3:**
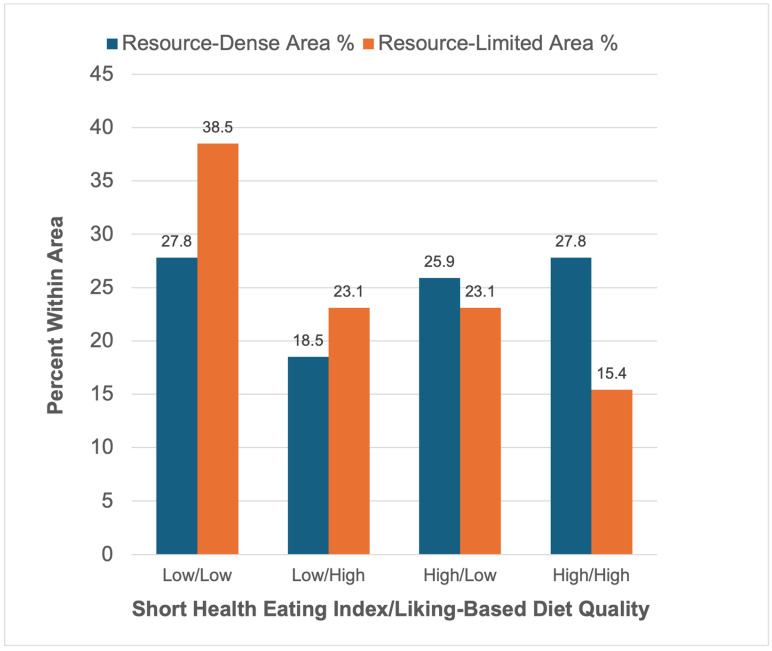
Distribution of diet quality groups by neighborhood context. The resource-dense area showed a relatively higher representation of the High sHEI/High DQI group, whereas the resource-limited area exhibited a greater concentration of participants in the Low sHEI/Low DQI group. Group distributions significantly differed by neighborhood context (χ^2^ = 25.70, *p* < 0.001).

Information from both diet quality indexes was used jointly to compare diet quality between the neighborhoods and is shown in Figure 3. The percentage was based on the total number of participants in each neighborhood. There were no differences in the distribution of groups in the resource-dense area. The highest proportions were Low DQI/Low sHEI (27.8%) and High DQI/High sHEI (27.8%) in the resource-dense area, indicating greater variability in the match between healthy dietary preferences and dietary quality intakes in a resource-rich context. By comparison, a higher percentage of the population in the resource-limited area belonged to the Low DQI/Low sHEI group (38.5%) and the lowest percentage in the High DQI/High sHEI group (15.4%). This suggests that a greater percentage of the population has consistently low dietary quality when physical resources are limited. The chi-square test was significant for the difference in the distribution of dietary concordance across the two neighborhoods (χ^2^ = 25.70, *p* < 0.001).

**Figure 4:**
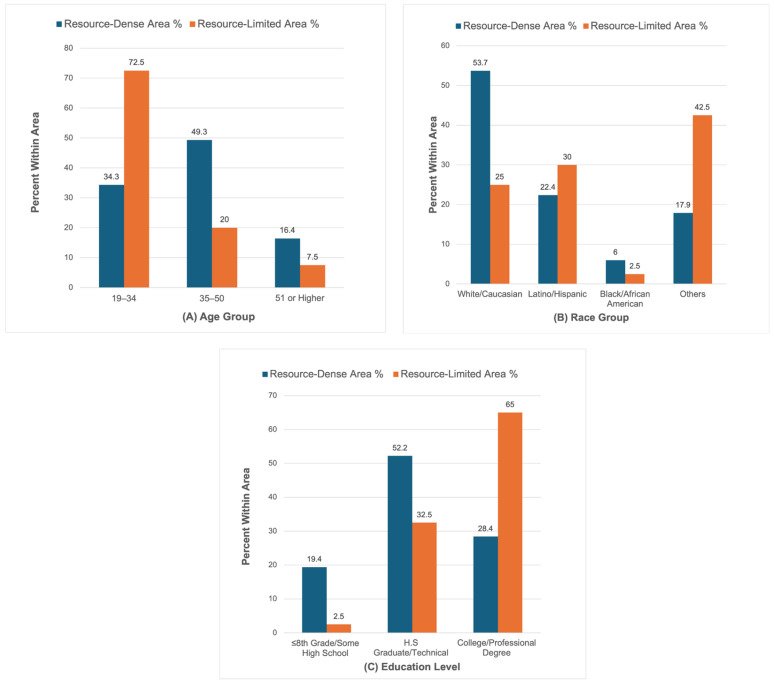
The descriptive comparisons of demographic distributions in resource-dense and resource-limited neighborhoods by (**A**) age, (**B**) race, and (**C**) education level. Percentages represent within-area distributions for each demographic group.

To better interpret neighborhood differences in these diet quality groups, descriptive analyses of the characteristics of the two neighborhood types were explored (Figure 4). These descriptions were used to inform interpretation of the diet quality groups rather than test primary outcomes.

As shown in Figure 4, there were differences across neighborhoods in terms of the demographic distributions. The age of 19 to 34 years had 72.5% of participants in the resource-limited area as opposed to 34.3% in the resource-dense area. Conversely, the resource-dense area was more likely to have more participants within the 35–50 age bracket (49.3% vs. 20.0%).

The composition of the races was also different. In the resource-dense area, the proportion of the White/Caucasian participants was larger than the resource-limited region (53.7% vs. 25.0%). The participants in the resource-limited area were also more Latino/Hispanic (30.0% vs. 22.4%).

The distribution of education levels was different between neighborhoods and not merely between categories. A significantly greater percentage of participants in the resource-limited area had a college or professional degree, 65.0% as opposed to 28.4% in the resource-dense area. In contrast, the resource-dense area contained more high-school/technical respondents (52.2%) and ≤8th-grade/some high-school (32.5%) respondents. In general, it was shown that the resource-limited area had greater educational attainment, although the categories were different.

Due to the significant differences in age, race, and education level between the two neighborhood contexts, the researchers used binary logistic regression to assess the relationship between the patterns of dietary concordance and the neighborhood context, holding other demographic variables constant. Table 3 demonstrates that in both unadjusted and adjusted models, participants in the High sHEI/High DQI group were more likely to live in the resource-dense area than the participants in the Low sHEI/Low DQI group (reference category). However, this association failed to be statistically significant on the adjustment of age, race, and level of education. The other concordance groups were not found to have any statistically significant associations.

## 4. Discussion

The present study examined nutrition vulnerability (NV) as a multidimensional tool of nutrition security to interpret neighborhood and spatial patterns of dietary inequity within communities, including structural, socioeconomic, behavioral, and environmental pressures [52,53,54]. This research developed and operationalized the NV tool by incorporating nutrition-related determinants and assessing their spatial patterns in a community. The Windham case study demonstrates the role of income constraints, access to transportation, and the capacity of residents to access and utilize resources all in the joint determination of nutritional vulnerability [16]. More recent spatial analyses have applied geographic exposure measures like density and distance of food outlets when analyzing food environments and obesity, showing that the large number of GIS-based approaches are still often centered on environmental availability and not on individual-level dietary and health measures [55]. In this framework, the NV Map is an analytic tool, which structures these interacting influences into a spatial format and enables the study of structural and behavioral constraints across neighborhoods without presuming that geographic distance to food outlets is the determining factor of nutritional conditions alone [56].

This NV tool comprised two measures of diet quality, use of food assistance, food security, and rates of obesity with only minimal inner correlation between these dimensions. Nonetheless, participation in food assistance (yes/no) was associated with greater frequency rates of obesity (ρ = 0.27, *p* < 0.01), thus suggesting that food assistance programs may either not reflect better nutritional outcomes [50] or that participation in food assistance may reflect societal vulnerability with limited structural and financial barriers to a healthy weight, according to a recent analysis of food assistance participants in Connecticut and Rhode Island [57].The existing literature has tended to indicate that SNAP beneficiation is correlated with better food security and a modest dietary advantage, without any consistent evidence of the elevated risk of obesity [58,59]. Food security showed a weak positive relationship with sHEI (ρ = 0.22, *p* < 0.01), indicating that higher food insecurity (i.e., higher scores representing low or very low food security) was associated with higher sHEI scores. Furthermore, the diet quality indicators did not significantly relate to rates of obesity in this sample. Recent systematic data have indicated that diet quality scores tend to be correlated with obesity and metabolic results among populations [60]. Interestingly, a recent analysis of the National Health and Nutrition Examination Survey did not find lower diet quality scores among obese adults, finding that adults may not perceive their diets as unhealthy, highlighting the need for health education on diet quality for the promotion of healthy weight and metabolic health [61]. Thus, the results in the present study imply that food availability, nutritional habits, and health are interrelated in a variety of ways, and one measure should not be viewed as a direct proxy of nutrition security.

The NV tool was examined across two regions of the community. The resource-dense and the resource-limited regions differed in retail density, transit coverage, and population composition. Differences in diet quality between these two regions were seen by leveraging information from both measures of diet quality, liking-based and frequency-based. Individuals with better diet quality from both measures were more frequent in the area with higher resources, whereas those with lower concordance were more frequent in the area with fewer resources. First, these findings highlight using multiple measures of diet quality is recommended to capture wider dimensions of dietary behavior that are not reflected in single-indicator comparisons [22]. Adults in the location with greater resources had a higher percentage in the High sHEI/High DQI category than the resource-limited area (27.8% vs. 15.4%, respectively). Conversely, the number of participants in the Low sHEI/Low DQI group was more concentrated within the resource-limited area as compared to the resource-dense one (38.5% vs. 27.8%). These results may reflect differences in the alignment between what is liked and the ability to access foods to fulfill these preferences across neighborhood contexts. According to the findings, with the help of the neighborhood resources, people can transform healthier food choices into the actual eating patterns. Nevertheless, it is not possible to access it only; it should be affordable, accompanied by nutrition education, and have management skills to handle food.

The diet quality differences diminished in the analysis adjusted for demographic differences, also suggesting the possibility of contextual or population-specific effects. This implies that mobility-based resources can aid in balancing geographic disadvantages on the neighborhood level [62]. High vehicle ownership rates in the younger population appear to compensate for the absence of nearby full-service grocery stores in the resource-limited area. This is reducing the incidences of obesity and slightly enhancing food security compared to what would be anticipated by considering residential food access alone [63,64,65]. The EPA National Walkability Index places both neighborhoods in the above-average to most-walkable range, which implies that there is no consistent low baseline built-environment walkability in either of the two neighborhoods. Nevertheless, practical access to food resources through walking might actually be different since (i) food outlets are not evenly spread in relation to residential blocks, portions with a resource constraint are out of the 0.5-mile radius commonly considered to assess urban food access, and (ii) the nature of routes such as hills or steep slopes can make them less perceived as walkable although they may have high walkability scores [66].

All these observations suggested that nutritional disadvantage in Windham is more influenced by the neighborhood conditions, which relate to the mobility of residents and their capacity to use resources rather than distance. The diet quality differences across regions of the community were attenuated supporting that age and race structure and education level were partially responsible for the differences observed between neighborhoods. These results clearly show that it is necessary to take into account the neighborhood population, along with the physical food environment. There was a major difference in education levels between neighborhoods, which provided an idea of the demographic composition of these trends: the increased education in the resource-limited area may suggest the existence of institution-based populations (including students and school staff) in the areas adjacent to university environments. In these settings, it is possible that demographically based eligibility requirements can determine the rates of participation in food assistance programs, rather than nutrition need itself. As an example, a range of university-related organizations, like international students, may not receive SNAP benefits, and it may be one of the reasons why these programs are less common in resource-dense areas [19]. This trend demonstrates that high educational capital may affect the nutritional and food safety levels of residents in the area; the mobility, information accessibility, and ability to choose food sources among this group of higher education individuals may all influence it. Although this was an exploration, this result supports the argument that demographic composition and physical food environments should be considered when explaining neighborhood-level NV. It proposes that food-access interventions would be enhanced by analyzing both the source of food and the movement of residents instead of depending on the residential food environment only [67,68,69].

Behavioral indicators of diet, food security status, obesity patterns, and SNAP participation combine in a single spatial analysis to enhance our capacity to identify how various disadvantage types aggregate at small geographic units [70]. This paper brings the individual-level nutrition data into the spatial environment, thus moving empirical approaches to NV beyond administrative or retailer-based measures alone [56,71].

## 5. Strength and Limitation

The main strengths of this study are the combination of individual-level nutrition and socioeconomic data with spatial analysis, several indicators to operationalize nutritional vulnerability, and the use of the concordance-based method to measure the correspondence of dietary preferences and actual behaviors.

The limitation of this study is that it falls under a cross-sectional design, which does not allow drawing conclusions regarding changes over time or the causal pathway of NV [72]. Spatial distributions in this case cannot be presumed to be long-term community trends. The census-block aggregation necessary to protect the confidentiality of the participants can obscure within-block variability and lower the accuracy of neighborhood-scale inferences [73]. Also, the convenience sampling method of considering community locations (e.g., food pantries, libraries, and community events) could lead to selection bias. The participants who were recruited in these environments might be systematically different from the overall community population in several aspects, such as their access to food, the program, and exposure to the community resources. Moreover, the recruiting patterns of space, where most of the sites were in the resource-dense area or close to it, might have affected the geographic distribution of recruits and, thus, the shown comparison at the neighborhood level. These are some of the factors that should be considered when generalizing the findings.

The dataset lacks several structural variables, like food affordability, food availability variability, cultural relevance of foods, and access to real-time transportation, which may also have an impact on dietary behavior but had not been measured in the current study. In addition, even though physical activity is a significant aspect of behavior that has the potential to interact with dietary habits, obesity and general nutritional susceptibility, this study did not measure the physical activity of participants. This missing variable could restrict the overall explanation of these health-related outcomes, especially the connection between the quality of diet and obesity. Also, the sample size is relatively low, especially in stratified analyses, which could limit statistical power and limit the ability to find some consistent differences in neighborhoods in subgroups.

Lastly, the Stunkard Figure Rating Scale (FRS) was used to measure obesity instead of objective anthropometric measurements. Even though FRS has been proven as a screening tool in the community-based setup, it can create bias of misclassification because body size can be under- or overestimated. This means that conclusions on obesity should be taken carefully. Although this is a limitation, it has been thought suitable in some community-based and resource-constrained research settings where direct anthropometric measures are not always feasible. Moreover, a visual scale might lessen the burden to the respondents and ease the participation in research, especially where underserved populations are involved.

## 6. Conclusions

This paper demonstrates that a fuller view of nutritional vulnerability (NV) in the Windham area can be achieved through the joint analysis of dietary quality, food security, obesity status, and food assistance data as a spatial analysis. These results support the idea that nutritional vulnerability is not related to the proximity only but is the product of demographic attributes, mobility trends, and the structural limits at the local level. The spatial patterns indicate that NV is highest in areas where the low socioeconomic status is combined with poor transport and access to food, which identifies the group that might be targeted by customized nutrition and mobility initiatives.

This article highlights the application of community-based dietary and socioeconomic data when mapped to provide analytical value. These local indicators reveal the trends that are not present in administrative data, which can contribute to the new research related to the association of structural and behavioral factors with nutrition equity. The analysis of these patterns allows visualizing patterns, which helps policymakers and community leaders prioritize the resources, design programs, and plan health interventions at the local level.

Future studies are recommended to expand the framework to include aspects like the affordability of food, access to transport, and real-time accessibility to enhance NV measurements. It is also suggested that behavioral determinants like physical activity be incorporated, as this would provide a more comprehensive picture of the interactions between diet, obesity, and overall health outcomes. The profile of food access in neighborhoods surrounding universities could be more comprehensive when activity-space approaches are incorporated, which are approaches that consider where people spend their time, out of their own neighborhoods. The use of the method across communities and over time would help determine whether there are patterns and whether they can be used to monitor the impacts of the policies. The NV Map can continue to be an evolving tool since it can assist local agencies and organizations in developing plans that not only promote nutritional equity but also help set up health resilience in resource-limited regions.

## Figures and Tables

**Table 1:** Spearman correlation coefficients (rho) among diet quality (DQI and sHEI), food security, obesity, and SNAP participation ^1^.

Variable Correlation Coefficient (Spearman Rho)	SNAP Participation Rate	Obese Rate	Food Security Level	Liking-Based DQI	sHEI Score
SNAP Participation Rate	1.00	0.27 **^,5^	−0.13	0.04	−0.12
Obese Rate		1.00	−0.12	−0.11	0.05
Food Security Level ^2^			1.00	0.10	0.22 **
Liking-Based DQI ^3^				1.00	0.11
sHEI Score ^4^					1.00

^1^ DQI and sHEI were derived from DFNL survey participants (*n* = 196), while FS, OB, and SNAP were computed from the merged dataset (*n* = 285). ^2^ Food security level was determined using the USDA six-item short-form food security module (score range: 0–6), with 0–1 being food secure, 2–4 indicating low food security, and 5–6 indicating very low food security. ^3^ Liking-based DQI = liking-based diet quality index, a diet quality index constructed from reported food/beverage likes and dislikes with higher scores indicating better diet quality [25]. ^4^ sHEI = short Healthy Eating Index, a condensed version of the USDA Healthy Eating Index constructed from a short food frequency survey with higher scores indicating better diet quality [26]. ^5^ Values represent Spearman rank-order correlation coefficients (ρ). Statistical significance (two-tailed) was set at α = 0.05; ** indicates *p* < 0.01.

**Table 2:** Sociodemographic, diet quality, and health characteristics of study participants in the different areas on NV Map (*n* = 107).

	Nutritional Vulnerability Map Areas		
Characteristic	% Participants in Resource-Dense Area (*n* = 67)	% Participants in Resource-Limited Area (*n* = 40)	*p* Value ^8,9^
**Age**			**<0.001**
19–34 (*n* = 52)	34.3 (23)	72.5 (29)	
35–50 (*n* = 41)	49.3 (33)	20.0 (8)	
51+ (*n* = 14)	16.4 (11)	7.5 (3)	
**Gender**			0.112
Men (*n* = 44)	44.8 (30)	35.0 (14)	
Women (*n* = 63)	55.2 (37)	65.0 (26)	
**Race and Ethnicity**			**<0.05**
White/Caucasian (*n* = 46)	53.7 (36)	25.0 (10)	
Latino/Hispanic (*n* = 27)	22.4 (15)	30.0 (12)	
Black/African American (*n* = 5)	6.0 (4)	2.5 (1)	
Others ^1^ (*n* = 29)	17.9 (12)	42.5 (17)	
**Education Level**			**<0.001**
≤8th Grade/Some High School (*n* = 14)	19.4 (13)	2.5 (1)	
H.S Graduate/Technical (*n* = 48)	52.2 (35)	32.5 (13)	
College/Professional Degree ^2^ (*n* = 45)	28.4 (19)	65.0 (26)	
**# of Drivable Vehicles ^2^**			**<0.001**
0 Vehicles (*n* = 49)	61.2 (41)	20.0 (8)	
≥1 Vehicles (*n* = 58)	38.8 (26)	80.0 (32)	
**BMI_Level**			0.827
Underweight (*n* = 5)	1.5 (1)	10.0 (4)	
Normal Weight (*n* = 36)	35.8 (24)	30.0 (12)	
Overweight (*n* = 41)	34.3 (23)	45.0 (18)	
Obese (*n* = 25)	28.4 (19)	15.0 (6)	
**Liking-Based DQI_Score ^3,4^**			0.771
Low (*n* = 24)	31.5 (17)	53.8 (7)	
Medium–Low (*n* = 13)	22.2 (12)	7.7 (1)	
Medium (*n* = 6)	9.3 (5)	7.7 (1)	
Medium–High (*n* = 5)	5.6 (3)	15.4 (2)	
High (*n* = 19)	31.5 (17)	15.4 (2)	
**sHEI Score ^4,5^**			0.147
Low (*n* = 20)	29.6 (16)	30.8 (4)	
Medium–Low (*n* = 4)	3.7 (2)	15.4 (2)	
Medium (*n* = 11)	16.7 (9)	15.4 (2)	
Medium–High (*n* = 7)	9.3 (5)	15.4 (2)	
High (*n* = 25)	40.7 (22)	23.1 (3)	
**Food Security Status ^6^**			0.646
Food Secure (0–1) (*n* = 40)	32.8 (22)	45.0 (18)	
Medium Food Insecurity (2–4) (*n* = 35)	35.8 (24)	27.5 (11)	
High Food Insecurity (5–6) (*n* = 32)	31.3 (21)	27.5 (11)	
**Food Assistance Program Participation Rate ^7^**			0.056
None (*n* = 57)	38.8 (26)	77.5 (31)	
≥1 Program (*n* = 50)	61.2 (41)	22.5 (9)	

^1^ Includes American Indian, Asian, Native Hawaiian or Other Pacific Islander, and Puerto Rican. ^2^ Based on the question: “How many drivable motor vehicles (cars, trucks, and motorcycles) are there in your household?”, # means number of drivable car. ^3^ Liking-based DQI with values ranging from −250 to 250; participants were categorized into quintiles based on the observed values. ^4^ DQI and sHEI were derived from DFNL survey participants (*n* = 196). ^5^ sHEI = short Healthy Eating Index (range: 0–100); participants were categorized into quintiles as DQI. ^6^ Assessed using a 6-item short form of the Household Food Security Scale. ^7^ Includes participation in major food assistance programs (e.g., SNAP, WIC, CSFP, CACFP, FMNP, SFMNP, and TEFAP). ^8^ Fisher’s exact test was used for analyses with small cell counts (expected frequency < 5). ^9^ All *p* values were obtained from regression-based models adjusted for age, race/ethnicity, and education level. Binary outcomes (e.g., food assistance participation, gender, and number of vehicles) were analyzed using multivariable logistic regression. Other outcomes were treated as continuous variables and analyzed using general linear models to facilitate adjusted comparisons across areas [48]. This approach is commonly applied when ordinal variables are used to approximate underlying continuous constructs [49,50,51].

**Table 3:** Unadjusted and adjusted odds ratios (ORs) for living in a resource-dense area (vs. resource-limited area) by dietary concordance groups among adult participants (*n* = 67) ^1,2^.

	Dietary Concordance Groups ^3,4^			
Neighborhood Context (Resource-Dense Area = 1)	Low/Low (*n* = 19)	Low/High (*n* = 14)	High/Low (*n* = 16)	High/High (*n* = 18)
Unadjusted	1	1.31 (0.26–6.72)	1.55 (0.31–7.81)	2.86 (0.48–17.11)
Adjusted	1	1.20 (0.20–7.11)	1.10 (0.18–6.73)	2.42 (0.35–16.81)

^1^ Odds ratios (ORs) were estimated using binary logistic regression models, with neighborhood context as the outcome (resource-dense area = 1, resource-limited area = 0). The Low sHEI/Low DQI group was used as the reference category. ^2^ Adjusted models controlled for age group, race, and education level. ^3^ Dietary concordance groups were derived by classifying DQI and sHEI scores based on their respective sample medians, resulting in four categories: Low sHEI/Low DQI, High sHEI/High DQI, Low sHEI/High DQI and High sHEI/Low DQI. ^4^ A total of *n* participants with complete sHEI and DQI data were included in these analyses. Participants with missing values for either dietary index were excluded.

## Data Availability

The original contributions presented in this study are included in the article. Further inquiries can be directed to the corresponding author.
